# Genome-wide association study for vascular aging highlights pathways shared with cardiovascular traits in Koreans

**DOI:** 10.3389/fcvm.2022.1058308

**Published:** 2022-12-22

**Authors:** JaeKyoung Ahn, Hankyeol Jeong, Bo-Gyeong Seo, Ki-Soo Park, Cheol Hwangbo, Han-Gyul Kim, Jin-Sin Koh, Jaemin Kim

**Affiliations:** ^1^Division of Applied Life Science (BK21 Four), Gyeongsang National University, Jinju, Republic of Korea; ^2^Institute of Agriculture and Life Sciences, Gyeongsang National University, Jinju, Republic of Korea; ^3^Division of Life Science, College of National Sciences, Gyeongsang National University, Jinju, Republic of Korea; ^4^Department of Preventive Medicine, College of Medicine and Institute of Health Science, Gyeongsang National University, Jinju, Republic of Korea; ^5^Center for Farmer’s Safety and Health, Gyeongsang National University Hospital, Jinju, Republic of Korea; ^6^Department of Internal Medicine, Gyeongsang National University School of Medicine, Gyeongsang National University Hospital, Jinju, Republic of Korea

**Keywords:** vascular aging, GWAS, endothelial dysfunction, prevention, cardiovascular disease

## Abstract

Vascular aging plays a pivotal role in the morbidity and mortality of older people. Reactive hyperemia index (RHI) detected by pulse amplitude tonometry (PAT) is a non-invasive measure of vascular endothelial function and aging-induced pathogenesis of both microvascular and macrovascular diseases. We conducted a genome-wide association study (GWAS) to comprehensively identify germline genetic variants associated with vascular aging in a Korean population, which revealed 60 suggestive genes underlying angiogenesis, inflammatory response in blood vessels, and cardiovascular diseases. Subsequently, we show that putative protective alleles were significantly enriched in an independent population with decelerated vascular aging phenotypes. Finally, we show the differential mRNA expression levels of putative causal genes in aging human primary endothelial cells *via* quantitative real-time polymerase chain reaction (PCR). These results highlight the potential contribution of genetic variants in the etiology of vascular aging and may suggest the link between vascular aging and cardiovascular traits.

## Introduction

Vascular aging is associated with the accumulation of reactive oxygen species (ROS) and chronic low-grade inflammation, which predispose to endothelial dysfunction and the development of atherosclerosis and stroke (1–4). In particular, oxidative stress causes the inactivation of endothelium-derived nitric oxide (NO), promoting age-related depletion in endothelium-mediated dilation, enhanced vasoconstriction, and impaired tissue perfusion (5, 6). Thus, it is important to establish a better mechanistic comprehension of the arterial aging process and evaluate both lifestyle and pharmacological countermeasures to treat this growing health issue (7, 8).

Members of families with exceptional longevity may have benefited from the enriched distribution of alleles that protect against diseases such as coronary artery disease (CAD), cancer, and type 2 diabetes, which ultimately contribute to population mortality (9, 10). While this hypothesis has been tested and supported by the identification of causal SNPs from genome-wide association studies (GWASs) showing robust associations with common diseases that are the main causes of death (11), the pursuit of genetic variants responsible for vascular aging to date has been greatly limited.

To navigate the spectrum of age-related vascular functional and phenotypic changes, we measured the reactive hyperemia index (RHI) in Korean individuals, an indicator of vascular endothelial function and cardiovascular risk factors of diabetes, high blood pressure frequency, and obstructive CAD (12–14). We aim to elucidate the novel pathways underlying vascular aging by conducting a multistage study, consisting of GWAS followed by genetic prediction in an independent population. We also test the plasma circulating cell-free DNA (cfDNA) for use in accurate genotyping in the case of the limited availability of a sufficient amount of genomic DNA (gDNA) which may be difficult to obtain for many clinical samples (15). Finally, we show the differential mRNA expression levels of putative causal genes in aging human primary endothelial cells which may further highlight targets for the prevention and control of aging-related vascular endothelial dysfunction and associated diseases. These results together highlight that GWAS in an underrepresented population in human genomics research, can lead to novel associations that merit future investigation and raise the possibility of using suggestive variants for risk prediction.

## Results

### Subject characteristics

We used the data from the Namgaram project consisting of a total of 1,010 subjects at the age of 50 or above, recruited from the southern site (Jinju) in Korea, all the individuals were measured for age-related qualitative and quantitative traits, including age, grip strength, muscle strength score, and vascular endothelial functions. Of these, 97 individuals were selected and genotyped using serum samples at 177,236 SNPs on Illumina Infinium SNP Genotyping Array ASA for the current study. We observed 95.8% overall genotype concordance between DNA extracted from gDNA (whole blood) and cfDNA (serum) across 19 overlapping individuals, indicating confidence in the accuracy of SNP calling from serum (Supplementary Table 1).

To assess the homogeneity of our cohort and to show that all subjects are of eastern Asian ancestry, the principal component analysis (PCA) was performed (Supplementary Figure 1). Multiple logistic regression analysis revealed that gender (*P* = 0.0348) and age (*P* = 0.0269) have statistically significant effects on endothelial vascular function (Supplementary Table 2). The endothelial vascular function tended to decrease in the elderly, and the trend was significantly stronger in women. The same analysis also showed a significant phenotypic correlation with age-related traits, loss of muscle (*P* = 0.0153), and grip strength (*P* = 0.0141). To carefully assess the association between genotypes and vascular aging, given the chronological age, we adjusted the phenotype for chronological age by simple regression and then standardized the residuals to *z*-scores, in each gender group separately.

### Identification of vascular aging-GWAS signals

**FIGURE 1:**
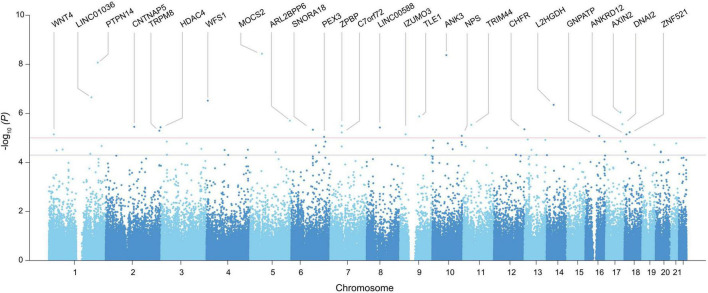
Manhattan plot of GWAS identified in the entire genome for vascular endothelial function. The *x*-axis represents the chromosome number, and the *y*-axis represents the *p*-value converted to -log. Only genes with SNPs exceeding the stringent significance threshold (*P* < 1.00E-05) were included in the plot, and the rest of the candidates above the nominal threshold (*P* < 1.00E-05) is provided in Table 1.

A GWAS including 97 Koreans was conducted using autosomal SNPs and RHI, adjusted for age, and standardized to *z*-scores in each gender group. A total of 64 SNPs which span 60 genes exceeded the suggestive genome-wide significance level of *P* = 5 × 10^–5^ (Figure 1 and Table 1). Three of these genes met the genome-wide significance level (*P* < 5 × 10^–8^). A genomic factor of 1.067 indicated no population structure in the cohort (Supplementary Figure 2). To confirm the robustness of the candidates, we additionally performed an association analysis by fitting age-related factors of grip strength and muscle mass as covariates, which still reproduced 54 significant genes out of 60 genes identified from the initial analysis (Supplementary Table 3). To test the bias of phenotypic resemblance among related individuals caused by non-genetic effects, we performed an association after the exclusion of one of the pair of individuals (estimated relationship > 0.05, *n* = 96), which retained 49 significant genes (Supplementary Table 4). We also executed GWAS from all SNPs imputed to the 1000 Genomes Project reference panel to increase statistical power (16), yet no additional genes reached the genome-wide significance (Supplementary Table 5).

**TABLE 1:** Genetic loci associated with vascular endothelial function after adjusting for age and sex.

Chr	bp	A1	A2	Freq	b	se	*p*	Closest gene
5	52398005	C	T	0.059	2.255	0.382	3.68E-09	*MOCS2*
10	62010155	T	C	0.494	-7.722	1.314	4.20E-09	*ANK3*
1	214659101	T	C	0.494	-7.575	1.315	8.46E-09	*PTPN14*
1	187209108	A	C	0.060	2.030	0.392	2.20E-07	*LINC01036*
4	6246543	C	T	0.489	-4.833	0.943	3.01E-07	*WFS1*
14	50724996	T	C	0.488	-4.777	0.946	4.45E-07	*L2HGDH*
17	63444990	C	T	0.051	2.015	0.410	9.07E-07	*AXIN2*
9	84302707	A	G	0.058	1.978	0.409	1.32E-06	*TLE1*
5	174611803	C	T	0.057	1.672	0.352	1.98E-06	*ARL2BPP6*
17	72314399	A	G	0.084	1.531	0.326	2.75E-06	*DNAI2*
11	35842967	A	G	0.108	1.282	0.274	2.93E-06	*TRIM44*
7	50152510	A	G	0.090	1.454	0.312	3.20E-06	*ZPBP*
2	125346013	A	G	0.076	1.714	0.370	3.53E-06	*CNTNAP5*
2	240156312	T	C	0.056	2.007	0.434	3.68E-06	*HDAC4*
8	58133642	C	T	0.091	1.409	0.305	3.76E-06	*LINC00588*
12	133473221	T	C	0.057	1.893	0.412	4.44E-06	*CHFR*
6	94922348	G	A	0.067	1.689	0.369	4.62E-06	*SNORA18*
2	234910442	C	T	0.065	1.671	0.366	5.07E-06	*TRPM8*
18	22800826	T	C	0.071	1.738	0.383	5.85E-06	*ZNF521*
7	50182079	T	C	0.092	1.473	0.325	5.98E-06	*C7orf72*
9	24618331	C	T	0.064	1.479	0.329	7.17E-06	*IZUMO3*
18	9158643	A	G	0.065	1.627	0.363	7.17E-06	*ANKRD12*
1	22472732	G	A	0.104	1.335	0.297	7.18E-06	*WNT4*
10	129381096	A	G	0.186	1.085	0.243	8.26E-06	*NPS*
16	60997653	T	C	0.081	1.441	0.323	8.37E-06	*GNPATP*
6	143797752	C	T	0.074	1.652	0.372	9.01E-06	*PEX3*
13	33757815	A	G	0.067	1.481	0.338	1.16E-05	*STARD13*
13	110633410	C	T	0.255	0.879	0.201	1.20E-05	*RN7SKP10*
10	6222774	A	G	0.062	1.599	0.367	1.29E-05	*PFKFB3*
17	63437097	T	C	0.085	1.444	0.332	1.37E-05	*AXIN2*
16	83979552	A	C	0.103	1.334	0.307	1.41E-05	*OSGIN1*
3	24632124	C	A	0.065	1.825	0.420	1.41E-05	*EIF3KP2*
6	149493632	A	G	0.198	1.021	0.235	1.42E-05	*TAB2*
10	127900223	A	C	0.082	1.522	0.351	1.49E-05	*ADAM12*
10	68371063	C	A	0.071	1.825	0.424	1.67E-05	*CTNNA3*
21	36797083	C	T	0.137	1.077	0.250	1.67E-05	*RUNX1*
3	111651285	A	G	0.223	0.882	0.205	1.71E-05	*PHLDB2*
10	130247163	G	A	0.124	1.283	0.300	1.89E-05	*MKI67*
19	53344701	G	A	0.066	1.544	0.361	1.91E-05	*ZNF28*
6	107764862	A	G	0.099	1.283	0.301	2.04E-05	*PDSS2*
1	231488541	A	G	0.070	1.786	0.420	2.14E-05	*SPRTN*
11	11317991	T	C	0.072	1.467	0.346	2.21E-05	*GALNT18*
7	50145007	A	G	0.090	1.393	0.328	2.23E-05	*ZPBP*
6	143793155	A	G	0.071	1.631	0.385	2.24E-05	*PEX3*
10	2916766	A	G	0.064	1.589	0.377	2.52E-05	*PFKP*
11	104461298	G	A	0.053	1.810	0.430	2.53E-05	*CASP12*
3	175798213	A	G	0.058	1.641	0.392	2.78E-05	*EI24P1*
10	89596013	A	G	0.070	1.755	0.420	2.89E-05	*CFL1P1*
1	60822886	G	A	0.134	1.109	0.266	2.98E-05	*PGBD4P8*
4	181536971	G	A	0.057	1.588	0.381	3.04E-05	*LINC00290*
13	49944406	C	T	0.058	1.782	0.427	3.06E-05	*CAB39L*
4	79906385	G	A	0.064	1.653	0.397	3.12E-05	*LINC01088*
1	34684463	G	A	0.158	0.970	0.233	3.21E-05	*C1orf94*
20	22772814	T	C	0.053	1.764	0.427	3.60E-05	*KRT18P3*
18	5439417	A	G	0.113	1.307	0.316	3.61E-05	*EPB41L3*
5	112179909	A	C	0.131	1.172	0.285	3.82E-05	*APC*
20	22746255	C	T	0.050	1.856	0.451	3.85E-05	*KRT18P3*
6	121559651	C	T	0.086	1.449	0.352	3.88E-05	*TBC1D32*
1	182038718	A	G	0.154	1.013	0.248	4.45E-05	*ZNF648*
3	25105548	T	C	0.054	1.580	0.389	4.85E-05	*RNA5SP125*
12	96563954	C	T	0.088	1.373	0.338	4.89E-05	*ELK3*
13	71789085	T	C	0.059	1.677	0.413	4.93E-05	*LINC00348*
4	95601206	T	C	0.123	1.273	0.314	4.96E-05	*PDLIM5*
9	111406348	T	C	0.063	1.485	0.366	4.98E-05	*RPL36AP35*

Genome-wide association study results with additional covariates of grip strength and muscle mass are provided in Supplementary Table 3. Chr, chromosome; Bp, physical position; A1, minor allele; A2, major allele; Freq, frequency of the minor allele; b, SNP effect; Se, standard error; *P*, *P*-value.

Using the initial GWAS results, we then defined the protective allele of each candidate SNP based on the directionality of association (β), as the allele responsible for the increase in the RHI value and thus decelerated vascular aging rates (Supplementary Table 6). We further screened these candidate genes for highly functional variants and identified missense variants in *MOCS2* and *SPRTN* genes (Supplementary Table 7), respectively, each involved in the synthesis of molybdenum auxiliary factors (17) and premature aging (18). In addition, the pathogenicity assessment of these variants, assessed by using the Combined Annotation-Dependent Depletion (CADD) database (19), is predicted to be in the top 0.9% (chr5:52398005, *MOCS2*) and 0.67% (rs78209580, *SPRTN*) of the most deleterious single-nucleotide substitutions that can be generated from the human genome. All 64 suggestive SNPs were also annotated to genes whose expression is associated with allelic variation at the variants level (*cis-*eQTLs) derived from publicly available data repositories, which additionally identified 15 candidate genes (Supplementary Table 8).

### Pathways putatively confer age-related vascular function

Over-representation analysis of gene ontology (GO) terms shows that vascular aging-associated genes are significantly enriched for functional categories including Wnt signaling pathway (GO:0016055) and negative regulation of canonical Wnt signaling pathway (GO:0090090), given the emerging roles of Wnt signaling in the regulation of angiogenesis, vasculature, and vascular diseases including atherosclerosis (20) (Table 2). By leveraging the human GWAS Catalog database and previously reported trait-associated loci (21), we found that “bilirubin measurement” and “bipolar disorder” traits consistently showed the most significant and robust enrichment (permutation *P* < 0.05) (Table 3 and Supplementary Table 9). Bilirubin plays a role as an effective antioxidant and is a preventive measure for the development of vascular diseases that can be mediated by vascular aging (19, 20). The tissue-specificity analysis further revealed the enrichment of associated genes in the pancreas, a key organ in diabetes mellitus as pancreatic β-cells are responsible for insulin biosynthesis and secretion (22).

**TABLE 2:** The significant gene ontology terms (*P* < 0.05) enriched from 60 genes putatively associated with vascular endothelial function.

Pathway	Term	*P*-value	Genes
GO:0016055	Wnt signaling pathway	7.35E-03	*TLE1, APC, AXIN2, WNT4*
GO:0031398	Positive regulation of protein ubiquitination	1.16E-02	*WFS1, SPRTN, CHFR*
GO:0001822	Kidney development	2.68E-02	*TBC1D32, WFS1, WNT4*
GO:0090090	Negative regulation of canonical Wnt signaling pathway	3.63E-02	*TLE1, APC, AXIN2*

**TABLE 3:** Summary of an enrichment of vascular endothelial function associated genes that overlap loci previously linked to phenotypes in GWAS studies.

Mapped trait	*N*	No. overlap	No. overlap from permutation	S.D. of No. overlap from permutation	*z*-score	*P*-value
Bilirubin measurement	974	30	2.11	7.8304	3.5616	3.69E-04
Bipolar disorder	608	10	2.01	2.3805	3.3560	7.91E-04
Eosinophil percentage of leukocytes	789	11	2.92	2.8357	2.8501	4.37E-03
Cytokine measurement	449	5	1.40	1.3468	2.6738	7.50E-03
Migraine disorder	354	5	1.12	1.9234	2.0172	4.37E-02

The *Z*-score represents the relative enrichment (or depletion if negative) of the number of the putative candidates overlapping loci linked to the corresponding trait relative to randomly selected genomic regions of the same length.

Based on the manual review of the previous literature and OMIM database, the candidate genes are related to the proliferation and differentiation of angiogenesis (*PTPN14*, *ELK3*, *ADAM12*, *WNT4*, *AXIN2*, *RNUX1*, *PFKFB3, PDLIM5*, and *EPB41L*) (23–31) and inflammatory responses in blood vessel system (*CASP12, PFKP, TLE1*, *HDAC4*, and *TAB2*) (32–36). Associated cardiovascular diseases include diabetes (*PDLIM5*, *HDAC4*, and *TLE1*) (31, 35, 37), cardiac development (*PDLIM5*) and myocardial aging (*CASP12*), hypertension (*PFKP* and *TAB2*) (31–33, 36), and intracerebral and intraventricular bleeding (*RUNX1*) and stroke (*AXIN2*) (24, 27).

### GWAS candidates can explain the attenuated vascular aging in the test population

**FIGURE 2:**
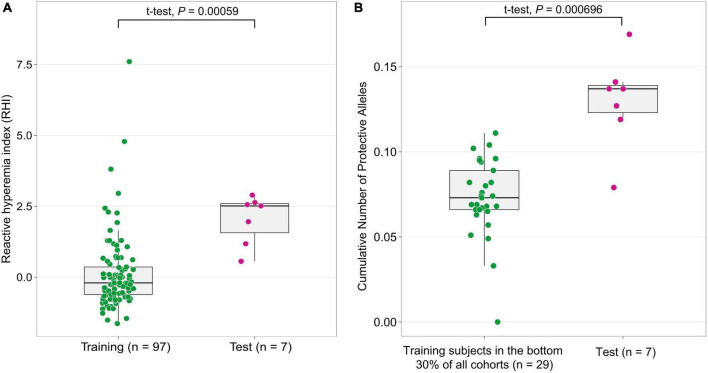
The cumulative effect of candidate genes on an independent subject population. **(A)** The independent test population was selected with subjects with standardized RHI values in the top 30% (*n* = 7, decelerated aging) of all Namgaram cohorts. **(B)** The distribution of the cumulative number of protective alleles in the test population was significantly higher (*P* < 1 × 10^–3^) than in the bottom 30% of all Namgaram cohorts (*n* = 29 with accelerated aging) selected in the training population.

To validate the GWAS findings and evaluate the cumulative effect of alleles from 64 candidate SNPs, we leveraged an independent sample of subjects (test population, *n* = 7) with standardized RHI values in the top 30% of all cohorts. The test population showed significantly higher standardized RHI values, indicative of attenuated vascular aging than the GWAS training subjects (*P* = 5.9 × 10^–4^, *t*-test) (Figure 2). We examined if the cumulative number of protective alleles of each subject in the test population is associated with elevated RHI values. We found that the distribution of protective alleles is significantly higher (*P* = 6.96 × 10^–4^) in the test population than in subjects in the bottom 30% of all Namgaram cohorts (*n* = 29 with accelerated aging) selected from the training population (Figure 2). The results together indicate that candidate loci have contributed a significant proportion of the marked difference in the rate of vascular aging.

### Differential expression of genes in aging vascular endothelium

**FIGURE 3:**
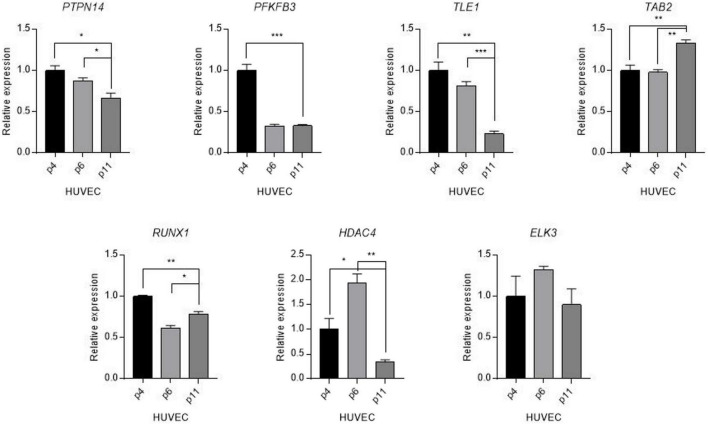
Differential expression of candidate genes in aged human vascular endothelium cells (HUVEC). Expression levels of candidate genes were quantified by using a real-time polymerase reaction chain (PCR) in human umbilical vein endothelial cells (HUVECs) of varying passages: young (p4), middle (p6), and old (p11). Values are presented as mean SEM. *p < 0.05, **p < 0.01, ***p < 0.001, n.s., not significant.

To understand the changes underpinning the heterogeneity of the aging process at a molecular level, we examined alterations in expression levels of candidate genes quantified by using real-time polymerase chain reaction (PCR) in human umbilical vein endothelial cells (HUVECs) of varying passages: relatively young (p4), middle (p6), and old (p11). The seven genes selected for this experiment were *PTPN14*, *TLE1*, *PFKFB3*, *TAB2*, *HDAC4*, *ELK3*, and *RUNX1*, chosen for their direct associations with vascular function and universally high expression level (Transcript per million, TPM > 100) in vascular tissues in GTEx database (38). We observed the most striking and significant decrease (75%) of *TLE1* mRNA expression level in aging human primary endothelial cells (*P* < 0.001) (Figure 3). Other tested genes also showed a significant age-related decrease (*PTPN14*, *PFKB3*, and *RUNX1*), increase (*TAB2*), and both (*HDAC4*) in gene expression variation.

### The ancestry-associated difference in *TLE1* protective allele frequency

**FIGURE 4:**
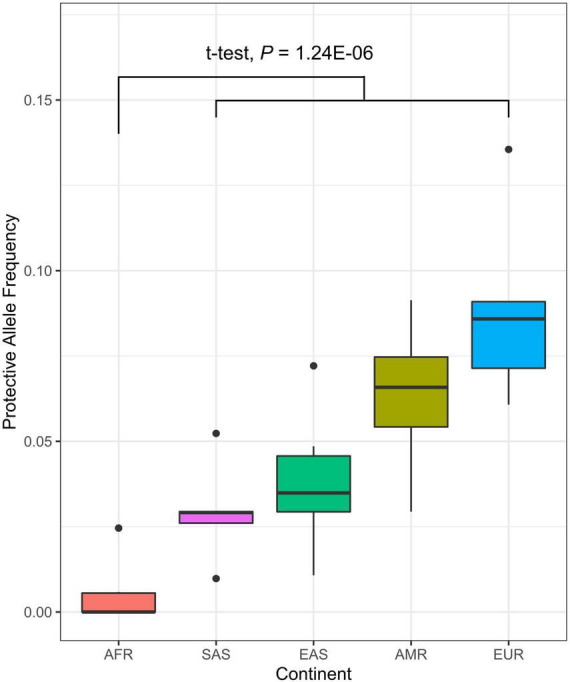
Distribution of protective allele frequency of *TLE1* gene (A allele; chr9:84302707) in diverse populations. Each represents the protective allele frequency of the corresponding country. The *t*-test describes the allele frequency differentiation between the African continent (AFR) and the rest (SAS, South Asia; EAS, East Asia; AMR, America; EUR, Europe).

The Centers for Disease Control and Prevention (CDC) has continuously reported the measures of disparity in the prevalence and incidence rate of medically diagnosed diabetes, with significantly higher diabetes risk in African black ancestry compared to other ethnic groups (39). To assess the risk imparted by candidate alleles in a diverse population, we utilized the publicly available genotypes of individuals from continent groups (Africa, South Asia, East Asia, America, and Europe) (40) on the seven candidate genes tested for differential expression for its association with diabetes. We tested the difference in allele frequency between ethnic groups and showed the significantly lower protective allele frequency in the *TLE1* gene in African-ancestry individuals (*P* = 1.24 × 10^–6^, *t*-test) (Figure 4 and Supplementary Table 10). Other significant genes with a consistent direction of lower protective allele frequency in African black ancestry also included *RUNX1* (*P* = 1.32 × 10^–5^), *PFKB3* (*P* = 1.41 × 10^–3^), and *PTPN14* (*P* = 1.75 × 10^–2^).

## Discussion

The GWAS of vascular aging in a sample of Korean revealed 60 associated genes, which provided added support for several previously reported associations with cardiovascular traits as well as some novel gene-based loci. At least 31 of these significant associations implicate genes with known or postulated roles in vascular function and cardiovascular diseases from the GWAS Catalog, supporting heightened investigations of pathways through which they are hypothesized to function (Supplementary Table 11). Of 31 GWAS catalog associations, we note that 15 and 13 are based on European and Asian populations, respectively. Given that 52% of GWAS have been conducted in populations of European ancestry, followed by 21% in Asians (41), our loci showed a disproportionately high number of genetic overlaps with reported associations from the same ethnicity. Strengths of our study thus include the extensively phenotyped epidemiologic cohorts of non-Europeans, the Namgaram cohort, and the novel association identified from the current study may address the lack of diversity in GWAS and enhance the understanding of the genetic contribution in the pathogenesis of vascular aging from the view of global health policy (42). In addition, the discovery of protective and risk alleles and their cumulative effects in vascular aging will be crucial in predicting the risk of complex traits, particularly in the Asian population, as inconsistencies in the directions of the effect of risk variants have been observed across ethnic groups (43).

Angiogenesis is both a crucial adaptive response to physiological stress and an endogenous regenerative response after ischemic injury, which contribute to the increased incidence of cardiovascular diseases in the elderly (44). The genes of the *ADAM* family play an important role in the blood vessel system, of which *ADAM12* is associated with angiogenesis and proliferation, and differentiation, and differences in expression within vascular smooth muscle cells were observed (26). *WNT4* is expressed in bone marrow mesenchymal stem cells (MSC) that can be differentiated into vascular endothelial cells (EC), and this expression of *Wnt4* is involved in regulating vascular regeneration (45). *PTPN14* expresses in multiple vascular (such as capillary or myocardial, pulmonary vein, and lung) endothelial cells and can regulate angiogenesis and vascular remodeling (46). Net (*ELK-3, Sap-2/ERP*) is one of the ternary complex factors that acts as an inhibitor of transcription. *ELK3* is included in the Net, and during mouse development, these Nets are observed in various parts and can be involved in and regulated angiogenesis (29). Most of the main energy production of endothelial cells occurs from glycolysis. *PFKFB3* is involved in this glycolysis to regulate angiogenesis. *PFKFB3* inhibition indicates a decrease in genes involved in aging, suggesting its importance in endothelial cells (47). *RUNX1* is essential for the development of hematopoietic and is importantly involved in blood and blood relationships. A study with *RUNX1* in Zebrafish ultimately resulted in death due to an abnormality in the blood vessels of the embryo and showed that DNA was similar in function to mammals or humans (27). *PDLIM5* plays a differential role in various organs and is associated with vascular remodeling and cardiomyopathy. AMPK is activated by hypoxia, ischemia, glucose loss, or stress, and is regulated by phosphorylation of cytoplasm. These AMPKs are important and involved in vascular and cardiovascular disease by phosphorylating *PDLIM5* in vascular smooth muscle cells to regulate cell migration (31). *EPH41L3* regulates cell–cell and cell–substratum interactions and cytoskeletal organization, controlling cell growth and differentiation (48).

Age-related activation of inflammatory processes plays a critical role in a broad range of macrovascular and microvascular pathologies (49). The expression of TLE1 is found in synovial sarcomas, and the *TLE* family can be a predictive marker for tumor diagnosis and a marker for treatment by indicating expression in various tumors (50). *TLE1* regulates the inflammatory response and regulates the path of NF-kB that controls immunity and cell self-destruction and development (34). TAB1 and *2* are proteins that bind to TAK1. TAK1 is affected by inflammatory cytokines and corresponding IL-1 or TNF leads to NF-kB activity. *TAB2*, expressed in human vascular endothelium, is associated with angiogenesis, and NF-kB, activated with inflammatory cytokines, is involved in cell fate and represents the importance of hematopoietic cell development (36, 51). Autophagy has a protective mechanism against inflammation and oxidative stress. Regulation of *PFKP* may contribute to autophagy regulation of vascular smooth muscle cells by partially engaging in PI3K-mTOR pathway activation in studies conducted in Dansam. Similarly, *HDAC4* has a significant relationship with autophagy and inflammation. Angiotensin II (Ang II) is the main medium of cardiovascular disease and is a phenotype that causes endothelial dysfunction. These *HDAC4*s, along with Ang II, are significantly involved in vascular inflammation in mice (33, 35). We also noted that two of the candidate genes, *EPB41L3* and *CASP12* showed age-dependent differential expression levels in the aging human and mouse cells, respectively (23, 32). Finally, although the molecular mechanisms of *SPRTN* in DNA translesional DNA synthesis, DNA replication, and ultimately accelerated aging have been elucidated (52), this novel association in vascular aging may provide new avenues for understanding fundamental cellular and molecular mechanisms of vascular aging upon further investigation.

A parsimonious explanation for the presence of many significant associations for a complex trait such as vascular aging is that the different associations are part of a high-order grouping of genes (53). Gene-set analysis additionally identified significant enrichment in the GWAS traits including bilirubin measurement and bipolar disorder. Bilirubin is the final product of hemoglobin metabolism and can act as an effective antioxidant in the body, and high levels of bilirubin are associated with the development and risk of cardiovascular disease and contribute to the protection of cell self-destruction or endothelial dysfunction due to aging (54, 55). The history of stroke in the presence of cardiovascular disease risk factors such as diabetes and hypertension is known to affect the risk of Alzheimer’s disease (56). Previous studies further reported evidence of genetic pleiotropy affecting cardiovascular disease and schizophrenia (57, 58), suggesting the genetic paths to other psychiatric disorders including bipolar disorder.

We hypothesized that the vascular aging phenotype can be attributed to transcriptional changes from the candidate genes identified in the current study. Of the seven genes investigated by quantitative real-time PCR, the mRNA level of the *TLE1* gene showed the strongest decrease in the aged HUVECs. Disrupted expression of TLE1 was observed in human type 2 diabetes and is associated with an increased proportion of glucagon-expressing cells (59). Considering that diabetes is a potent risk factor for most geriatric syndromes (60), the dysregulation of TLE1 in older passages can reflect the aging process. Given that overexpression of TAB2 suppresses autophagy (61), coupled with the evidence that autophagic activity declines with age in diverse organisms (62), we observed an increase in TAB2 expression in the aging HUVECs. While *HDAC4* was identified as a gene with an upward expression during the aging process of the mouse (63), we observed a contradictory result. Additional investigation is required to test if this gene represents the difference in the developmental trajectories of expression across different species (64).

Given that researchers and clinicians often face the problem of acquiring enough high-quality DNA for microarray or next-generation sequencing analysis, the high concordance rate between genotypes called from gDNA of whole blood and cfDNA of serum demonstrates that this method provides a reliable approach for the use with a common SNP genotyping array and application in GWAS. However, as previously reported (65), we note that there is a decrease in the total genotype call rates with cfDNA extracted from serum/plasma.

Compared with the previous GWAS, the present study made novel contributions to (1) understand the genetic landscape of vascular aging *via* the quantitative trait of RHI in the underrepresented population; (2) examine the out-of-sample prediction ability of GWAS results; and (3) conduct functional *in vitro* expression analysis with candidate genes. However, our study should be received in the context of its limitations; first, the sample size was restricted by the small number of subjects. The consequences of this include the overestimates of effect size and low reproducibility of results (66). However, the relevance of genes with known cardiovascular traits and endothelial functions, the replication analysis, and the *in vitro* expression experiment together provide support and putative mechanisms for the involvement of these genes in vascular aging. Second, it is worth mentioning that the Korean cohort data used in this study were sampled from a particular cohort with a specific age range. Since genetic ancestries are known as common confounding effects, one should be cautious to generalize these findings to general populations or particular clinical cohorts. We attempted to expand our findings by incorporating the genomes of other ethnic groups and provided the one of possible genetic mechanisms behind the ethnic disparities in diabetes. With the addition of data from diverse genetics studies, future research will be required to successfully address these limitations and advance our biological understanding of vascular aging.

In conclusion, we showed by the GWAS and replication of the association followed by the functional examination of candidate genes which will serve as an important repository for future GWAS of vascular aging. A thorough experimental investigation of suggestive genes may provide possible clues to a biological mechanism that can at least partially underlie the common genetic architecture of age-related cardiovascular diseases.

## Materials and methods

### Ethics statement

All participants provided written informed consent. This study was approved by the Institutional Review Board of Gyeongsang National University (approval number: GIRB-A16-Y-0012).

### Study design and phenotype

This cross-sectional study used data from the Namgaram cohort. The cohort consisted of a group of people living in rural communities who were aged 50 years or older and all agreed to participate in this cohort from 2015 to 2017. All 1,010 subjects enrolled in the Namgaram cohort answered questionnaires and underwent physical examinations, blood tests, and radiographic examinations. To specifically infer the vascular endothelial function, we used a non-invasive EndoPAT device, to measure the pulse signal amplitude at the fingertip, quantified as a RHI value (67). We adjusted the phenotype of RHI for the age effect using the model, *y* = b0 + b1 × age + e, and then standardized the residuals to *z*-scores in each gender group separately by using the “lm” function in R software (68).

### Genomic data and quality control

Genomic and circulating cell-free DNAs were extracted from 26 whole blood and 97 serum samples, respectively. For cfDNA, Whole Genome Amplification (WGA) was performed using a REPLI-g Qiagen kit following the protocol in the manufacturer’s manual. The DNAs were then genotyped by a high-density Illumina Infinium Asian Screening Array (ASA-24v1). We excluded SNPs with missingness > 20% (“–geno 0.20”) and minor allele frequency (MAF) < 0.05 (“–maf 0.05”) and retained a total of 177,236 autosomal SNPs (GRCh37) by using PLINK 1.9 (69). The genotype data had been imputed to 1000 Genomes panel using IMPUTE2 software (70). After applying the same quality control criteria, there were 3,084,757 autosomal genotyped/imputed SNPs. The data presented in the study are deposited in the Figshare repository, at https://doi.org/10.6084/m9.figshare.21323802.v2.

### Genome-wide association study

The linear model implemented in the GCTA tool (71) was used to test for association with vascular aging phenotypes. We first estimated the genetic relationship matrix (GRM) between all pairs of individuals from all the genotyped SNPs (“–make-grm”) and then tested for the effect of each SNP on the phenotypes (“–mlma” option). The same analysis was run with the inclusion of grip strength and muscle mass as covariates (“–qcovar”) to test for the robustness of the initial GWAS results. In addition, we excluded one of each pair of individuals with an estimated relationship > 0.05 to avoid the possibility that the phenotypic resemblance between close relatives could be because of non-genetic effects (for example, shared environment) (72). The SNPs that achieved a *P* = 5 × 10^–5^ were considered statistically suggestive and were annotated to the closest genes. SnpEff was used to predict the deleterious effect of each candidate SNP (73).

### Gene set enrichment analysis

DAVID 6.8^1^ (74) was used to determine if there was significant enrichment of genes with specific functional categories (GO) among GWAS candidate genes. A *p*-value of 0.05 was employed as the criterion for statistical significance. GWAS Catalog (75) was also used to find significant over-representation of particular GWAS traits. The traits with at least 300 previously reported associated hits were retained for the analysis. The number of 60 candidate genes overlapping the GWAS variant was calculated. To determine if the number of overlaps for each GWAS trait was more extreme than expected, the same number of regions of the same sizes were resampled from the genome 1000 times. Taken together, we estimated the *z*-score, associated *P*-value, and false discovery rate (FDR) using the “pnorm” and “adjust” functions of R software (68). Five independent permutations were repeated to test the robustness of the result.

### Tissue-specific expression

The Genotype-Tissue Expression (GTEx) V8^2^ database (38) was used to characterize the expression landscape of each candidate gene across all available human tissues. FUMA GWAS^3^ (76) (“SNP2FUNC” function) was used to identify the tissue specificity of candidate genes. In addition, the “SNP2GENE” function was used to annotate significant SNPs to genes based on eQTL mapping by applying a FDR of 0.05 to limit the results to significant variant gene pairs.

### Ancestry associated allele

To assess the allele frequency in a more diverse population (Africa, America, Europe, East Asia, and South Asia), we utilized the 1000 genome project (77). Leveraging the whole-genome data, PCA was further conducted using the GCTA tool (“pca 20”) (71), and the figure was generated by the first two principal components.

### Statistical analyses and visualization

Statistical analyses were assessed by *t*-test to determine the difference in allele frequency distribution between continental groups and RHI values between discovery training and the test populations using R software (68). A Manhattan plot and boxplots were generated using “ggplot2,” “ggpubr,” “smplot,” and basic built-in packages of the same software.

### Measurement of mRNA level by quantitative real-time PCR

The human endothelial cells (HUVECs) were purchased from Gibco and cultured in Medium 199 (sigma, M4530) supplemented with 20% FBS (GenDEPOT, F0900-050), 30 μg/ml of endothelial cell growth supplement (ECGS) (corning, 306006) and 100 μg/ml heparin (sigma, H3149). Total RNA was extracted using NucleoSpin RNA kit (macherey-nagel, 740955) following the manufacturer’s methods. Extracted RNAs were synthesized into cDNA using the iScript cDNA Synthesis kit (bio-rad, BR1708891). Quantitative real-time polymerase chain reaction (q-PCR) was performed using the IQ SYBR Green Supermix (bio-rad, BR1708882) with Rotor-Gene Q-Pure Detection system (QIAGEN). The primer list used for quantitative real-time polymerase chain reaction is provided in Supplementary Table 12. The gene expression was quantified relative to the reference gene (GAPDH). At least three replicates were measured for each group. Statistical significance was assessed by one-way analysis of variance (ANOVA) using GraphPad Prism software v7.00 (GraphPad) were used to test the significance of the data. *P*-values of < 0.05 were considered statistically significant.

## Data availability statement

The data presented in this study are deposited in the Figshare repository, at 10.6084/m9.figshare.21323802.v2.

## Ethics statement

All participants provided written informed consent. This study was approved by the Institutional Review Board of Gyeongsang National University (approval number: GIRB-A16-Y-0012). The patients/participants provided their written informed consent to participate in this study.

## Author contributions

JK, J-SK, H-GK, and K-SP conceived and designed all of the described experiments. B-GS and CH contributed to the qPCR. JA and HJ analyzed the data. JA, J-SK, and JK drafted the manuscript. All authors read and approved the final manuscript.

## Footnotes

1
https://david.ncifcrf.gov/tools.jsp

2
https://gtexportal.org

3
https://fuma.ctglab.nl
