# Supplementary material for: Genome-wide association study for vascular aging highlights pathways shared with cardiovascular traits in Koreans
Source: Front Cardiovasc Med. 2022 Dec 22;9:1058308. doi: 10.3389/fcvm.2022.1058308 (PMC9813851; doi:10.3389/fcvm.2022.1058308)

**Figure. S1 Principal Component Analysis (PCA) of East Asian and Korean cohort.** The PCA comparison between East Asians and Koreans explains that all Korean individuals have no significant structural difference from East Asian descent.


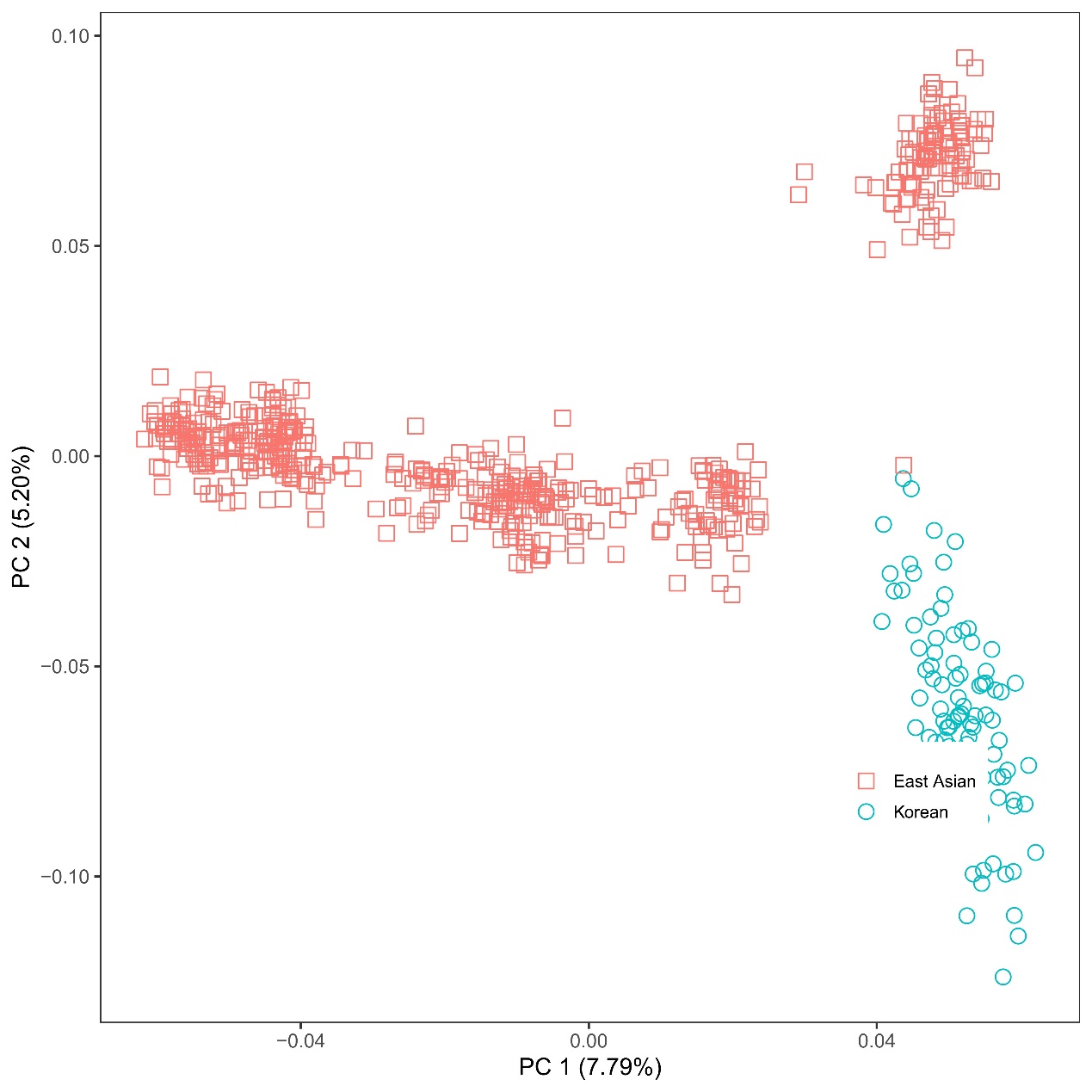


**Fig. S2 Quantile-quantile (Q-Q) plot of p values identified in the entire genome.** The red line describes the expected distribution (expected p-value) and the observed distribution (observed p-value) between the SNP and the phenotype. lambda = 1.067


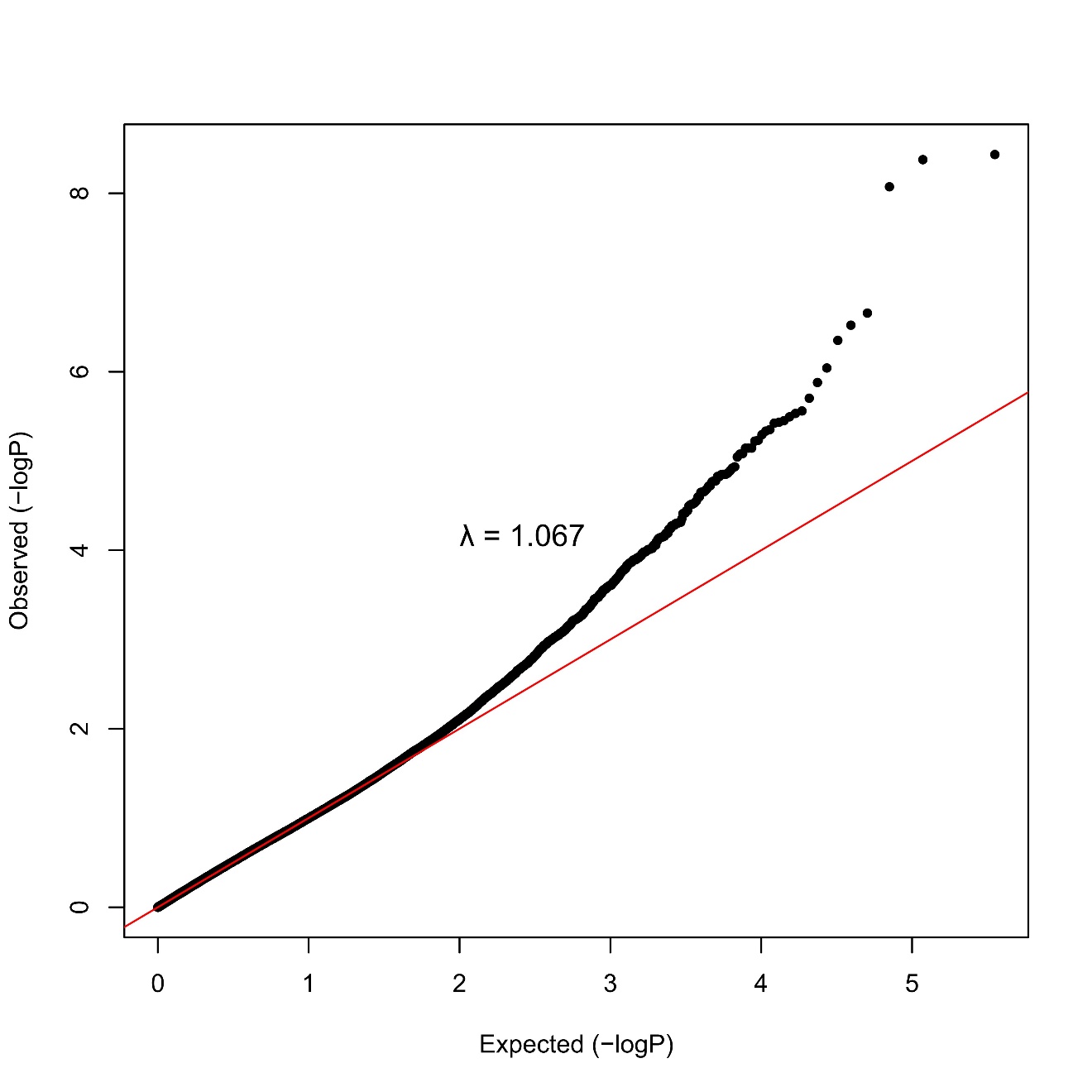

Supplement: Supplementary file 1 [file Data_Sheet_1.docx]
